# Academic Goals, Student Homework Engagement, and Academic Achievement in Elementary School

**DOI:** 10.3389/fpsyg.2016.00463

**Published:** 2016-03-31

**Authors:** Antonio Valle, Bibiana Regueiro, José C. Núñez, Susana Rodríguez, Isabel Piñeiro, Pedro Rosário

**Affiliations:** ^1^Department of Developmental and Educational Psychology, University of A CoruñaA Coruña, Spain; ^2^Department of Psychology, University of OviedoOviedo, Spain; ^3^Departmento de Psicologia Aplicada, Universidade do MinhoBraga, Portugal

**Keywords:** homework, academic goals, student homework engagement, approach to homework, academic achievement, elementary school

## Abstract

There seems to be a general consensus in the literature that doing homework is beneficial for students. Thus, the current challenge is to examine the process of doing homework to find which variables may help students to complete the homework assigned. To address this goal, a path analysis model was fit. The model hypothesized that the way students engage in homework is explained by the type of academic goals set, and it explains the amount of time spend on homework, the homework time management, and the amount of homework done. Lastly, the amount of homework done is positively related to academic achievement. The model was fit using a sample of 535 Spanish students from the last three courses of elementary school (aged 9 to 13). Findings show that: (a) academic achievement was positively associated with the amount of homework completed, (b) the amount of homework completed was related to the homework time management, (c) homework time management was associated with the approach to homework, (d) and the approach to homework, like the rest of the variables of the model (except for the time spent on homework), was related to the student's academic motivation (i.e., academic goals).

## Introduction

Literature indicates that doing homework regularly is positively associated with students' academic achievement (Zimmerman and Kitsantas, 2005). Hence, as expected, the amount of homework done is one of the variables that shows a strong and positive relationship with academic achievement (Cooper et al., 2001).

It seems consensual in the literature that doing homework is always beneficial to students, but it is also true that the key for the academic success does not rely on the amount of homework done, but rather on *how* students engage on homework (Trautwein et al., 2009; Núñez et al., 2015c), and on *how* homework engagement is related with student motivation (Martin, 2012). There is, therefore, a call to analyze the process of homework rather than just the product; that is, to examine the extent to which the *quality* of the process of doing homework may be relevant to the final outcome.

## Trautwein's model of homework

The model by Trautwein et al. (2006b) is rooted in the motivational theories, namely the theory of the expectancy value (Eccles (Parsons) et al., 1983; Pintrich and De Groot, 1990), and the theory of self-determination (Deci et al., 2002), as well as on theories of learning and instruction (Boekaerts, 1999). Trautwein and colleagues' model analyzes students' related variables in two blocks, as follows: the motivational (aiming at directing and sustaining the behavior) and the cognitive and behavioral implications (cognitions and behaviors related to the moment of doing homework).These two blocks of variables are rooted in the literature. Motivational variables are related with the theory of expectancy-value by Eccles (Parsons) et al. (1983), while the variables addressing students' implication are related with the school engagement framework (e.g., Fredricks et al., 2004). However, as Eccles and Wang (2012) stress, both models are interrelated due to the fact that both variables are closely related and show reciprocal relationships.

## Student homework engagement: the interplay between cognitive and behavioral components

Engagement is a relatively new construct with great relevance in the field of psychology and instruction (Fredricks et al., 2004). Generally considered, engagement has been described as the active implication of the person in an activity (Reeve et al., 2004). However, despite the close relation between engagement and motivation, literature clearly differentiates between them (e.g., Martin, 2012), stressing engagement as the behavioral manifestation of motivation (Skinner and Pitzer, 2012), or arguing that motivation is a precursor of engagement rather than part of it. In sum, motivation relates to the “why” whereas the engagement focuses on the “what” of a particular behavior.

**Figure 1:**
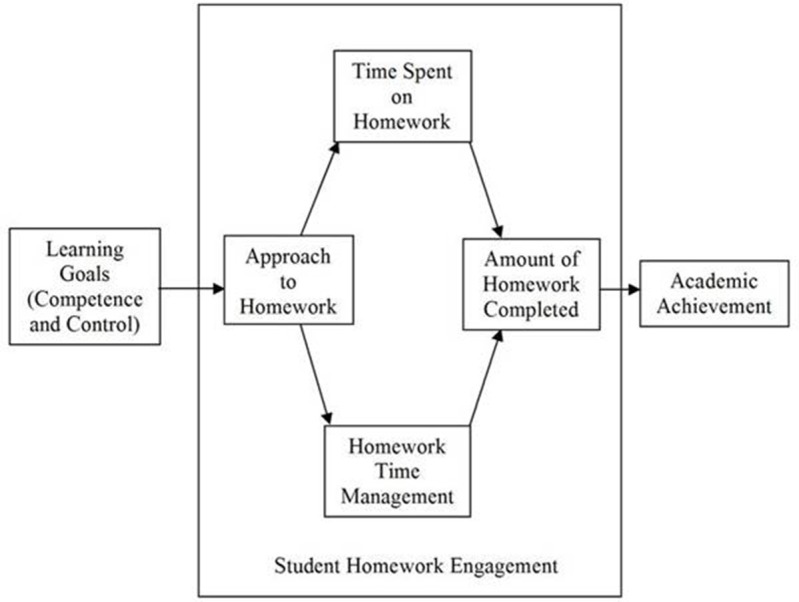
**General model hypothesized to explain the relationship between academic motivation, student homework engagement, and academic achievement**.

Consistent with this perspective, the current research fitted a model with the variable engagement mediating the relationship between motivation and academic achievement (see Eccles and Wang, 2012). Engagement is a complex construct with observational and non-observational aspects (Appleton et al., 2008). Some researchers conceptualize engagement with two dimensions—behavior and emotions (e.g., Marks, 2000)—while others define engagement with four dimensions—academic, behavioral, cognitive, and emotional (e.g., Appleton et al., 2006). In the current study, we followed Fredricks' et al. (2004) conceptualization of engagement as a construct with three dimensions: cognitive (e.g., approaches to learning), behavioral (e.g., student homework behaviors), and emotional (e.g., interest, boredom). For the purpose of the present study, the dimension of emotion was not included in the model (see Figure 1).

### Cognitive homework engagement

In the past few decades, a robust body of research has been addressing the relationship between the way students deal with their learning process and academic outcomes (Marton and Säljö, 1976a,b; Struyven et al., 2006; Rosário et al., 2010a, 2013a). Marton and Säljö (1976a,b) examined how students studied an academic text and found two ways of approaching the task: a surface and a deep approach. The surface approach is characterized by learning the contents aiming at achieving goals that are extrinsic to the learning content. In contrast, the deep approach is characterized by an intrinsic interest in the task and students are likely to be focused on understanding the learning content, relating it to prior knowledge and to the surrounding environment (Entwistle, 2009; Rosário et al., 2010b). The metaphor “surface vs. deep” constitutes an easy to perceive conceptual framework, both in the classroom setting and in other educational settings (i.e., doing homework at home), and has been shown to be a powerful tool for parents, teachers, and students when conceptualizing the ways students approach school tasks (Entwistle, 1991; Rosário et al., 2005). The core of the concept of approaches to studying (or to learning) is the metacognitive connection between an intention to approach a task and a strategy to implement it (Rosário et al., 2013b).

The process of doing homework focuses on what students do when completing homework, that is, how they approach their work and how they manage their personal resources and settings while doing homework. It is likely that students' approaches to homework may influence not only the final homework outcome but also the quality of that process. Students who adopt a deep approach are likely to engage their homework with the intention of deepening their understanding of the knowledge learned in class. In this process, students often relate the homework exercises to prior knowledge and monitor their mastery of the content learned. This process involves intrinsic intention to understand the ideas and the use of strategies to build meaning (Cano et al., 2014). In contrast, students who approach homework with a surface approach are likely to do homework with extrinsic motivation (e.g., rewards of their parents, fear of upsetting their teacher). Their goals may target finishing homework as soon as and with the less effort possible to be able to do more interesting activities. Students using this approach are more likely to do homework to fulfill an external obligation (e.g., hand in homework in class and get a grade), than for the benefits for learning.

### Behavioral homework engagement

Findings from prior research indicate that the more the implication of students in doing their homework the better the academic achievement (Cooper et al., 2006). Following Trautwein et al. (2006b), our conceptualization of student homework engagement includes behaviors related with the amount of homework done, time spent on homework, and homework time management (e.g., concentration). In the present investigation, these three variables were included in the model (see Figure 1).

Extant findings on the relationship between the amount of homework done and academic achievement are in need of further clarification. Some authors argue for a strong and positive relationship (e.g., Cooper et al., 2006), while others found that this relationship is higher throughout schooling (Cooper et al., 2001; Zimmerman and Kitsantas, 2005). Authors explained this last finding arguing that the load of homework assigned by teachers vary throughout schooling, and also that the cognitive competencies of students are likely to vary with age (Muhlenbruck et al., 2000). More recently, Núñez et al. (2015c) found that the relationship between these two variables varied as a function of the age of the students enrolled. Particularly, this relationship was found to be negative in elementary school, null in junior high school, and positive in high school.

Moreover, the relationship between the amount of homework done and academic achievement relates, among other factors, with the students' age, the quality of the homework assigned, the type of assessment, and the nature of the feedback provided. For example, some students may always complete their homework and get good grades for doing it, which does not mean that these students learn more (Kohn, 2006). In fact, more important than the quantity of the homework done, is the quality of that work (Fernández-Alonso et al., 2014).

Another variable included in the model was the time spent on homework. Findings on the relationship between time spent on homework and academic achievement are mixed. Some studies found a positive relationship (Cooper et al., 2001, 2006) while others found a null or a negative one (Trautwein et al., 2006b, 2009). In 2009, Dettmers, Trautwein and Lüdtke conducted a study with data from the PISA 2003 (Dettmers et al., 2009). Findings on the relationship between the number of hours spent on homework and academic achievement in mathematics show that the students in countries with higher grades spend fewer hours doing homework than students in countries with low academic grades. At the student level, findings showed a negative relationship between time spent on homework and academic achievement in 12 out of 40 countries.

The relationship between the amount of homework done, time dedicated to homework, and academic achievement was hypothesized to be mediated by the homework time management. Xu (2007) was one of the pioneers examining the management of the time spent on homework. Initially, Xu (2007) did not find a relationship between time management and academic achievement (spend more time on homework is not equal to use efficient strategies for time management). Latter, Xu (2010) found a positive relationship between students' grade level, organized environment, and homework time management. More recently, Núñez et al. (2015c) found that effective homework time management affects positively the amount of homework done, and, consequently, academic achievement. This relationship is stronger for elementary students when compared with students in high school.

## Academic motivation and student homework engagement relationship

Literature has consistently shown that a deep approach to learning is associated positively with the quality of the learning outcomes (Rosário et al., 2013b; Cano et al., 2014; Vallejo et al., 2014). The adoption of a deep approach to homework depends on many factors, but students self-set goals and their motives for doing homework are among the most critical motivational variables when students decide to engage in homework.

Literature on achievement motivation highlights academic goals as an important line of research (Ng, 2008). In the educational setting, whereas learning goals focus on the comprehension and mastery of the content, performance goals are more focused on achieving a better performance than their colleagues (Pajares et al., 2000; Gaudreau, 2012).

Extant literature reports a positive relationship between adopting learning goals and the use of cognitive and self-regulation strategies (Elliot et al., 1999; Núñez et al., 2013). In fact, students who value learning and show an intention to learn and improve their competences are likely to use deep learning strategies (Suárez et al., 2001; Valle et al., 2003a,b, 2015d), which are aimed at understanding the content in depth. Moreover, these learning-goal oriented students are likely to self-regulate their learning process (Valle et al., 2015a), put on effort to learn, and assume the control of their learning process (Rosário et al., 2016). These students persist much longer when they face difficult and challenging tasks than colleagues pursuing performance goals. The former also use more strategies oriented toward the comprehension of content, are more intrinsically motivated, and feel more enthusiasm about academic work. Some researchers also found positive relationships between learning goals and pro-social behavior (e.g., Inglés et al., 2013).

Reviewing the differentiation between learning goals and performance goals, Elliot and colleagues (Elliot and Church, 1997; Elliot, 1999; Elliot et al., 1999) proposed a three-dimensional framework for academic goals. In addition to learning goals, performance goals were differentiated as follows: (a) performance-approach goals, focused on achieving competence with regard to others; and (b) performance-avoidance goals, aimed at avoiding incompetence with regard to others. Various studies have provided empirical support for this distinction within performance goals (e.g., Wolters et al., 1996; Middleton and Midgley, 1997; Skaalvik, 1997; Rodríguez et al., 2001; Valle et al., 2006). Moreover, some authors proposed a similar differentiation for learning goals (Elliot, 1999). The rationale was as follows: learning goals are characterized by high engagement in academic tasks, so an avoidance tendency in such goals should reflect avoidance of this engagement. Hence, students who pursue a work avoidance goal are likely to avoid challenging tasks and to put on effort to do well, only doing the bare minimum to complete the task. In general, learning goals are associated with a large amount of positive results in diverse motivational, cognitive, and achievement outcomes, whereas performance goals have been linked to less adaptive outcomes, or even to negative outcomes (Valle et al., 2009).

## Aims of this study

Several relationships between motivational, cognitive, and behavioral variables involving self-regulated learning in the classroom have recently been studied (Rosário et al., 2013a). However, there is a lack of knowledge of the relationships between these variables throughout the process of doing homework.

The principal purpose of this work (see Figure 1) is to analyze how student homework engagement (cognitive and behavioral) mediates motivation and academic performance. This study aims to provide new information about an issue that is taken for granted, but which, as far as we know, lacks empirical data. The question is: to what extent students acknowledge homework as a good way to acquire competence, improve their skills and performance? Our working hypothesis is that student value homework in this regard. Therefore, we hypothesized that the more students are motivated to learn, the more they will be involved (cognitively and behaviorally) in their homework, and the higher their academic achievement.

To address this goal, we developed a path analysis model (see Figure 1) in which we hypothesized that: (a) the student's motivational level is significantly related to their cognitive homework engagement (i.e., the approach to studying applied to homework), and their behavioral homework engagement (i.e., amount of time spent and homework time management, and amount of homework completed); (b) student's cognitive and behavioral homework engagement are positively associated with academic achievement; and (c) cognitive and behavioral homework engagement are related (the more deep cognitive engagement, the more time spent and time management, and the more amount of homework is done).

## Methods

### Participants

The study enrolled 535 students, aged between 9 and 13 (*M* = 10.32, *SD* = 0.99), of four public schools, from the last three years of the Spanish Elementary Education (4th, 5th, and 6th grade level), of whom 49.3% were boys. By grade, 40.4% (*n* = 216) were enrolled in the 4th grade, 35.1% (*n* = 188) in the 5th grade, and 24.5% (*n* = 131) in the 6th grade.

### Measures

#### Learning goals

The level and type of motivation for academic learning was assessed with the Academic Goals Instrument (Núñez et al., 1997). Although, this instrument allows differentiating a broad range of academic goals, for the purposes of this work, we only used the subscale of *learning goals* (i.e., competence and control). The instrument is rated on a 5-point Likert-type scale, with responses ranging from one (not at all interested) to five (absolutely interested in learning and acquiring competence and control in the different subjects). An example item is: “I make an effort in my studies because performing the academic tasks allows me to increase my knowledge.” The reliability of the scale is good (α = 0.87).

#### Approach to homework

To measure the process of approaching homework, we adapted the *Students' Approaches to Learning Inventory* (Rosário et al., 2010a, 2013a), taking into account both the students' age and the homework contexts. This instrument is based on voluminous literature on approaches to learning (e.g., Biggs et al., 2001; Rosário et al., 2005), and provides information about two ways of approaching homework. For the purpose of this research, we only used the deep approach (e.g., “Before starting homework, I usually decide whether what was taught in class is clear and, if not, I review the lesson before I start”). Students respond to the items on a 5-point Likert-type scale ranging from one (not at all deep approach) to five (completely deep approach). The reliability of the scale is good (α = 0.80).

#### Time spent on homework, homework time management, and amount of homework completed

To measure these three variables, we used the Homework Survey (e.g., Rosário et al., 2009; Núñez et al., 2015a,b; Valle et al., 2015b,c). To measure the *time spent on homework*, students responded to three items (in general, in a typical week, on a typical weekend) with the general formulation, “How much time do you usually spend on homework?,” with the response options 1, <30 min; 2, 30 min to 1 h; 3, 1 h to an hour and a half; 4, 1 h and a half to 2 h; 5, more than 2 h. Homework time management was measured through the responses to three items (in general, in a typical week, on a typical weekend) in which they were asked to indicate how they managed the time normally spent doing homework, using the following scale: 1, I waste it completely (I am constantly distracted by anything); 2, I waste it more than I should; 3, regular; 4, I manage it pretty much; 5, I optimize it completely (I concentrate and until I finish, I don't think about anything else). Finally, the *amount of homework completed* by students (assigned by teachers) was assessed through responses to an item about the amount of homework usually done, using a 5-point Likert-type scale (1, none; 2, some; 3, one half; 4, almost all; 5, all).

#### Academic achievement

Assessment of academic achievement was assessed through students' report card grades in Spanish Language, Galician Language, English Language, Knowledge of the Environment, and Mathematics. Average achievement was calculated with the mean grades in these five areas.

### Procedure

Data of the target variables was collected during regular school hours, by research assistants, after obtaining the consent of the school administration and of the teachers and students. Prior to the application of the questionnaires, which took place in a single session, the participants were informed about the goals of the project, and assured that data was confidential and used for research purposes only.

### Data analysis

The model was fit with AMOS 18 (Arbuckle, 2009). The data were previously analyzed and individual cases presenting a significant number of missing values were eliminated (2.1%), whereas the rest of the missing values were replaced by the mean. Taking into account the analysis of the characteristics of the variables (e.g., skewness and kurtosis in Table 1), we used the maximum likelihood method to fit the model and estimate the values of the parameters.

**Table 1:** **Means, standard deviations, skewness, kurtosis, and correlation matrix of the target variables**.

	**1**	**2**	**3**	**4**	**5**	**6**
1. Learning goals	–					
2. Approach to homework	0.50^**^	–				
3. Amount of homework done	0.42^**^	0.33^**^	–			
4. Time spent on homework	−0.01	−0.03	0.10^*^	–		
5. Time management	0.45^**^	0.45^**^	0.39^**^	−0.02	–	
6. Academic achievement	0.43^**^	0.13^**^	0.34^**^	−0.01	0.24^**^	–
*M*	4.26	4.02	4.28	2.41	3.77	3.21
*SD*	0.74	0.80	0.63	1.05	0.97	1.02
Skewness	−1.26	−0.89	−1.10	0.37	−0.67	−0.13
Kurtosis	1.05	0.62	1.29	−0.72	−0.10	−0.56

* *p < 0.05*.

** *p < 0.01*.

A series of goodness-of-fit statistics were used to analyze our model. Beyond chi-square (χ^2^) and its associated probability (*p*), the information provided by the goodness-of-fit index (GFI) and the adjusted goodness-of-fit index (AGFI; Jöreskog and Sörbom, 1983); the comparative fit index (CFI) (Bentler, 1990); and the root mean square error of approximation (RMSEA; Browne and Cudeck, 1993) was used. According to these authors, the model fits well when GFI and AGFI > 0.90, CFI > 0.95, and RMSEA ≤ 0.05.

## Results

### Descriptive analysis

The relations between the variables included in the model as well as the descriptive statistics are shown in Table 1. All the variables were significantly and positively related, except for the time spent on homework, which was only related to the amount of homework done. According to the value of the means of these variables, students in the last years of elementary school: (a) reported a high level of motivation to learn and mastery; (b) used preferentially a deep approach to homework; (c) did the homework assigned by the teachers most of the times; (d) usually spent about an hour a day on homework; (e) reported to manage their study time effectively; and (f) showed a medium-high level of academic achievement.

### Evaluation and re-specification of the initial model

**Figure 2:**
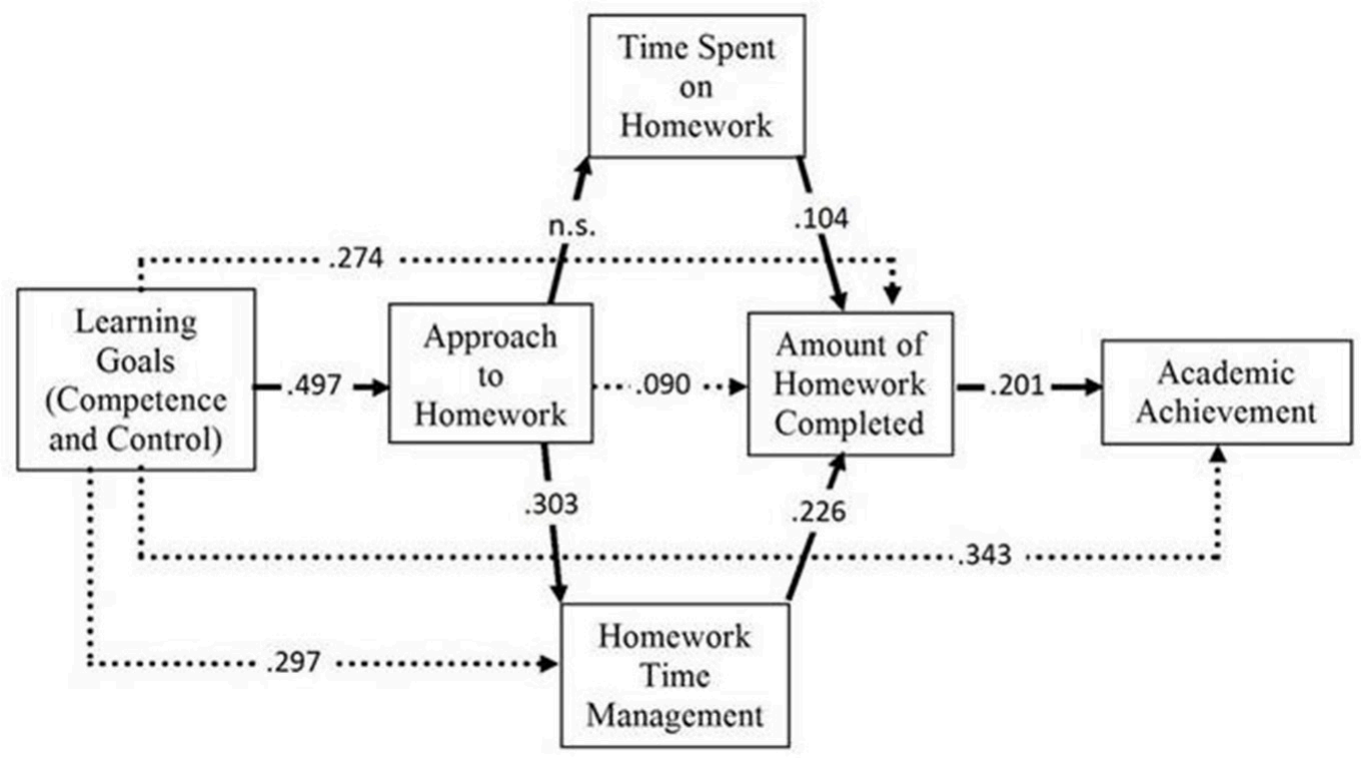
**The results of the fit of the hypothesized model (standardized outcomes): Relations in dashed lines were found to be statistically significant, but this was not established in the initial model**.

The data obtained indicated that the initial model (see Figure 1) presented a poor fit to the empirical data: χ^2^ = 155.80, *df* = 8, *p* < 0.001, *GFI* = 0.917, *AGFI* = 0.783, *TLI* = 0.534, *CFI* = 0.751, *RMSEA* = 0.186, 90% CI (0.161, 0.212), *p* < 0.001. Analysis of the modification indexes revealed the need to include three direct effects initially considered as null, and to eliminate a finally null effect (included in the initial model as significant). The strategy adopted to modify the initial model involved including and estimating the model each time a new effect was included. The final model comprised three effects (academic goals on homework time management, on amount of homework done, and on academic achievement) and the elimination of the initially established effect of the approach to studying on the time spent doing homework. The inclusion or elimination of the effects in the model was determined accounting for their statistical and theoretical significance. The final model resulting from these modifications is shown in Figure 2, with an adequate fit to the empirical data: χ^2^ = 12.03, *df* = 6, *p* = 0.061, *GFI* = 0.993, *AGFI* = 0.974, *TLI* = 0.975, *CFI* = 0.990, *RMSEA* = 0.043, 90% CI (0.000, 0.079), *p* = 0.567.

### Assessment of the relationships on the final model

Table 2 presents the data obtained for the relationships considered in the final model (see also Figure 2).

**Table 2:** **Fit of the hypothesized model (standardized outcomes): final model of student engagement in homework**.

	***b*^a^**	***b*^b^**	**SE**	**CR**	***P*<**
Learning goals → Approach to homework	0.536	0.497	0.040	13.248	0.001
Approach to homework → Time-management	0.350	0.303	0.049	7.093	0.001
Learning goals → Time-management	0.370	0.297	0.053	6.960	0.001
Time-management → Amount of homework	0.179	0.226	0.035	5.143	0.001
Learning goals → Amount of homework	0.270	0.274	0.045	6.054	0.001
Time spent on homework → Amount of homework	0.067	0.104	0.024	2.768	0.006
Approach to homework → Amount of homework	0.082	0.090	0.042	1.974	0.048
Amount of homework → Academic achievement	0.310	0.201	0.065	4.763	0.004
Learning goals → Academic achievement	0.521	0.343	0.064	8.128	0.202

a Nonstandardized regression coefficients;

b *Standardized regression coefficients*.

The data from Table 2 and Figure 2 indicates that the majority of the relationships between the variables are consistent with the hypotheses. First, we found a statistically significant association between the learning goals (i.e., competence and control), the approach to homework (*b* = 0.50, *p* < 0.001), two of the variables associated with engagement in homework (the amount of homework done [*b* = 0.27, *p* < 0.001], homework time management [*b* = 0.30, *p* < 0.001]), and academic achievement (*b* = 0.34, *p* < 0.001). These results indicate that the more oriented students are toward learning goals (i.e., competence and control), the deeper the approach to homework, the more homework is completed, the better the homework time management, and the higher the academic achievement.

Second, a statistically significant association between the deep approach and homework time management (*b* = 0.30, *p* < 0.001) and the amount of homework done (*b* = 0.09, *p* < 0.05) was found. These results reflect that the deeper the students' approach to homework, the better the management of the time spent on homework, and the more the homework done. Third, there was a statistically significant association between homework time management, time spent on homework, and the amount of homework done (*b* = 0.23, *p* < 0.001, and *b* = 0.10, *p* < 0.01, respectively). These results confirm, as expected, that the more time students spent doing homework and the better students manage their homework time, the more homework they will do. Four, we found a statistically significant relation between the amount of homework done and academic achievement (*b* = 0.20, *p* < 0.001). This indicates that the more homework students complete the better their academic achievement.

In summary, our findings indicate that: (a) academic achievement is positively associated with the amount of homework completed; (b) the amount of homework done is related to homework time management; (c) homework time management is associated with *how* homework is done (approach to homework); and (d) consistent with the behavior of the variables in the model (except for the time spent on homework), how homework is done (i.e., approach to homework) is explained to a great extent (see total effects in Table 3) by the student's type of academic motivation.

**Table 3:** **Standardized direct, indirect, and total effects for the final model**.

**—(direction of the effect) →**	**Approach to homework**	**Time management**	**Amount of homework completed**	**Academic achievement**
**STANDARDIZED OUTCOMES**				
Academic goals	0.497	0.297	0.274	0.343
Approach to homework	–	0.303	0.090	0.000
Time spent on homework	0.000	0.000	0.104	0.000
Time management	0.000	–	0.226	0.000
Amount of homework done	0.000	0.000	–	0.201
**STANDARDIZED INDIRECT EFFECTS**				
Academic goals	0.000	0.150	0.146	0.084
Approach to homework	–	0.000	0.068	0.032
Time spent on homework	0.000	0.000	0.000	0.021
Time management	0.000	–	0.000	0.046
Amount of homework done	0.000	0.000	–	0.000
**STANDARDIZED TOTAL EFFECTS**				
Academic goals	0.497	0.447	0.420	0.428
Approach to homework	–	0.303	0.158	0.032
Time spent on homework	0.000	0.000	0.104	0.021
Time management	0.000	–	0.226	0.046
Amount of homework done	0.000	0.000	–	0.201

Finally, taking into account both the direct effects (represented in Figure 2) and the indirect ones (see Table 3), the model explained between 20 and 30% of the variance of the dependent variables (except for the time spent on homework, which is not explained at all): approach to homework (24.7%), time management (26.9%), amount of homework done (24.4%), and academic achievement (21.6%).

## Discussion

Consistent with prior research (e.g., Cooper et al., 2001), our findings showed that students' academic achievement in the last years of elementary education is closely related to the amount of homework done. In addition, the present study also confirms the importance of students' effort and commitment to doing homework (Trautwein et al., 2006a,b), showing that academic achievement is also related with students' desire and interest to learn and improve their skills. Therefore, when teachers assign homework, it is essential to attend to students' typical approach to learning, which is mediated by the motivational profile and by the way students solve the tasks proposed (Hong et al., 2004). The results of this investigation suggest that the adoption of learning goals leads to important educational benefits (Meece et al., 2006), among which is doing homework.

Importantly, our study shows that the amount of homework done is associated not only with the time spent, but also with the time management. Time spent on homework should not be considered an absolute indicator of the amount of homework done, because students' cognitive skills, motivation, and prior knowledge may significantly affect the time needed to complete the homework assignment (Regueiro et al., 2015). For students, managing homework time is a challenge (Corno, 2000; Xu, 2008), but doing it correctly may have a positive influence on their academic success (Claessens et al., 2007), on homework completion (Xu, 2005), and on school achievement (Eilam, 2001).

Despite, that previous studies reported a positive relationship between the time spent on homework and academic achievement (Cooper et al., 2006), the present research shows that time spent on homework is not a relevant predictor of academic achievement. Other studies have also obtained similar results (Trautwein et al., 2009; Núñez et al., 2015a), indicating that time spent on homework is negatively associated to academic achievement, perhaps because spending a lot of time on homework may indicate an inefficient working style and lack of motivation (Núñez et al., 2015a). Besides, our data indicates that spending more time on homework is positively associated to the amount of homework done.

Although, some studies have found that students who spend more time on homework also tend to report greater commitment to school work (Galloway et al., 2013), our findings indicated that spending more time doing homework was not related to a deeper engagement on the task. A possible explanation may be that using a deep approach to school tasks subsumes engaging in homework with the aim of practicing but also to further extend the content learned in class. This approach does not depends on the time spent doing homework, rather on the students' motives for doing homework.

Another important contribution of this study concerns learning-oriented goals—usually associated with positive outcomes in motivational, cognitive, and achievement variables (Pajares et al., 2000). Results indicate that the motivation to increase competence and learning is also related to approaching homework deeply and to manage homework efficiently. Consistent with previous findings (Xu, 2005), these results provide additional empirical support to time management goals (Pintrich, 2004).

There is a robust relationship between learning-oriented goals and a deep approach, and between a deep approach and the amount of homework done. All this indicates that these results are in line with prior research, meaning that the adoption of a deep approach to learning is related with high quality academic achievement (Lindblom-Ylänne and Lonka, 1999; Rosário et al., 2013b).

### Educational implications and study limitations

One of the major limitations of this study lies in the type of research design used. We used a cross-sectional design to examine the effects among the variables within a path analysis model. However, to establish a cause-effect relationship a temporal sequence between two variables is needed a requirement that can only be met with longitudinal designs. Future studies should consider address this limitation.

Despite the above limitation, our results can be considered relevant and show important educational implications. It is essential for teachers and school administrators to be sensitized about the effects of teachers' homework follow-up practices on students' homework engagement (Rosário et al., 2015), and of these variables in students' school engagement and academic success. Likewise, research on students' learning should be undertaken from the perspective of the learners to understand how students use their knowledge and skills to do homework and to solve problems posed therein. On the other hand, research should examine in-depth the use of learning strategies during homework, as well as how students' motivations at an early age may foster homework completion and increase the quality of school outcomes. For this last purpose, teachers should pay attention not only to the acquisition of curricular content but also to the development of the appropriate thinking skills and self-regulated learning strategies (Rosário et al., 2010b; Núñez et al., 2013). Finally, the amount of homework done and its positive relationship with academic achievement should be considered as a final outcome of a process rooted on a comprehensive and meaningful learning. Students motivated to learn are likely to approach homework deeply and manage homework time efficaciously. As a result, they tend to do more homework and outperform. In sum, is doing homework a good way to acquire competence, improve skills, and outperform? Our data suggest a positive answer.

## Author contributions

AV and BR Collect data, data analysis, writing the paper. JN and PR data analysis, writing the paper. SR and IP writing the paper.

### Conflict of interest statement

The authors declare that the research was conducted in the absence of any commercial or financial relationships that could be construed as a potential conflict of interest.
